# A prototype photoplethysmography-based cuffless device shows promising results in tracking changes in blood pressure

**DOI:** 10.3389/fmedt.2024.1464473

**Published:** 2024-10-21

**Authors:** Christine Hove, Frode Wirum Sæter, Alexey Stepanov, Kasper Gade Bøtker-Rasmussen, Trine M. Seeberg, Espen Westgaard, Sondre Heimark, Bård Waldum-Grevbo, Jonny Hisdal, Anne Cecilie K. Larstorp

**Affiliations:** ^1^ Institute of Clinical Medicine, University of Oslo, Oslo, Norway; ^2^ Department of Nephrology, Oslo University Hospital, Oslo, Norway; ^3^ Department of Vascular Surgery, Oslo University Hospital, Oslo, Norway; ^4^ Aidee Health AS, Bærum, Norway; ^5^ Section for Cardiovascular and Renal Research, Oslo University Hospital, Oslo, Norway; ^6^ Department of Medical Biochemistry, Oslo University Hospital, Oslo, Norway

**Keywords:** cuffless, blood pressure, healthy adults, machine learning, blood pressure changes

## Abstract

**Introduction:**

Non-invasive cuffless blood pressure devices have shown promising results in accurately estimating blood pressure when comparing measurements at rest. However, none of commercially available or prototype cuffless devices have yet been validated according to the appropriate standards. The aim of the present study was to bridge this gap and evaluate the ability of a prototype cuffless device, developed by Aidee Health AS, to track changes in blood pressure compared to a non-invasive, continuous blood pressure monitor (Human NIBP or Nexfin) in a laboratory set up. The performance was evaluated according to the metrics and statistical methodology described in the ISO 81060-3:2022 standard. However, the present study is not a validation study and thus the study was not conducted according to the ISO 81060-3:2022 protocol, e.g., non-invasive reference and distribution of age not fulfilled.

**Method:**

Data were sampled continuously, beat-to-beat, from both the cuffless and the reference device. The cuffless device was calibrated once using the reference BP measurement. Three different techniques (isometric exercise, mental stress, and cold pressor test) were used to induce blood pressure changes in 38 healthy adults.

**Results:**

The mean difference (standard deviation) was 0.3 (8.7) mmHg for systolic blood pressure, 0.04 (6.6) mmHg for diastolic blood pressure, and 0.8 (7.9) mmHg for mean arterial pressure, meeting the Accuracy requirement of ISO 81060-3:2022 (≤6.0 (10.0) mmHg). The corresponding results for the Stability criteria were 1.9 (9.2) mmHg, 2.9 (8.1) mmHg and 2.5 (9.5) mmHg. The acceptance criteria for the Change requirement were achieved for the 85th percentile of ≤50% error for diastolic blood pressure and mean arterial pressure but were higher than the limit for systolic blood pressure (56% vs. ≤50%) and for all parameters for the 50th percentile (32%–39% vs. ≤25%).

**Conclusions:**

The present study demonstrated that the cuffless device could track blood pressure changes in healthy adults across different activities and showed promising results in achieving the acceptance criteria from ISO 81060-3:2022.

## 1 Introduction

Cuffless, wearable blood pressure (BP) measurement devices (cuffless devices) have the potential to provide continuous, beat-to-beat BP estimations during daily routines, without significant discomfort to the user (1, 2). Despite considerable research in this field, the accuracy of cuffless devices remains uncertain (3). In these devices BP is estimated by device specific models, using the input from physiological variables and signals that are related to changes in BP. Most of them use pulse wave analysis of photoplethysmographic (PPG) signals, pulse arrival time (PAT) or a combination of both (4).

Several cuffless devices have been shown to accurately predict BP in subjects at rest under controlled conditions in the laboratory (5–8). Some cuffless devices are even commercialized as validated according to the European Society of Hypertension International Protocol Revision 2010 (5) and/or ISO 81060-2:2019 protocol (6, 9, 10), which are not intended for cuffless devices. There are several issues with validation of cuffless devices using these protocols.

First, they are designed to test intermittent automated cuff-based BP devices during static conditions over a short period of time. In contrast to the cuff-based devices which aim to measure the actual pressure, the cuffless BP devices provide surrogate BP estimations from non-pressure signals and are prone to fluctuations in these signals which are not related to BP (3, 11, 12).

Second, most cuffless BP devices rely on an initial, individual calibration that is usually performed at rest using a standard cuff-based BP device. Essentially these devices track changes in BP relative to the calibration value (13). In a stable, resting condition BP variations are small, and might be almost negligible, especially when the duration of protocol is short. In these cases, the device would seem to track BP accurately, but this does not guarantee the same performance over longer periods of time or under substantial BP changes (14).

Third, various situations commonly encountered in daily life, such as physical activity, mental stress, and perception of physical pain, produce changes in BP through different physiological mechanisms. Thus, devices used in clinical evaluation of BP must be able to accurately estimate BP changes from a variety of activities (3).

To address these issues, several standards and recommendations have been published recently. The ISO 81060-3:2022 standard (15) focuses on validation of cuffless, noninvasive BP devices that provide continuous, beat-to-beat or high-resolution BP estimations. On the other hand, the ESH 2023 recommendations (3) describe the validation procedure for intermittent cuffless devices for use in ambulatory settings. To the best of our knowledge, no cuffless device has yet been validated according to either the ISO 81060-3:2022 standard or to the ESH 2023 recommendations (3).

In contrast to most studies which evaluated the performance of cuffless devices at rest, the aim of the present study was to address these limitations and use three well-known techniques to alter BP by different physiological mechanisms [isometric exercise (16), mental stress (17, 18), and cold pressor test (19–21)] to investigate the ability of a prototype, PPG-based cuffless device, placed on the upper arm, to track BP compared to a non-invasive, continuous BP monitor in healthy adults. The present pilot study was designed as part of the development of the cuffless device (Aidee Health AS, Norway), towards a future validation. Thus, the performance of the cuffless device was evaluated according to the metrics from the ISO 81060-3:2022 standard (ISO 3).

## 2 Materials and methods

### 2.1 Participants

Healthy volunteers ≥18 years of age, free of any chronic or cardiovascular disease, were eligible for inclusion. Potential participants were screened with a short interview, BP measurements (inclusion BP) and a 12-lead electrocardiogram (ECG). Candidates with pregnancy, inclusion BP ≥ 180/120 mmHg or any contraindication to standard cardiac stress testing (22) were excluded. In line with the Helsinki declaration (23), all participants were informed about the test procedure and signed a written informed consent form before inclusion. The participants were instructed to avoid intake of any food during the two hours prior to the test, as well as caffeine drinks and nicotine during the four hours before the test and alcohol at any time on the day of the test. During the test the participants were dressed in comfortable clothes to minimally interfere with the experimental conditions.

The study was approved by the Regional committees for medical and health research ethics (REK, Norway, project number 65844) prior to the inclusion of the first participant.

### 2.2 Reference blood pressure

Reference BP was measured continuously and non-invasively by the volume-clamp method using either Human NIBP Nano System (AD Instruments, Sydney, Australia) or Nexfin (24–30) (Bmeye, Amsterdam, The Netherlands). Two different reference BP devices were used as the Nexfin device malfunctioned during the study and was replaced with the Human NIBP, which uses the same measurement principle. The parent technology, Finapres (FMS Finapres, Medical systems BV, Amsterdam) has been validated for research use (31–33) and is commonly accepted for non-invasive BP measurements in non-critically ill patients (17, 34, 35). The finger pressure cuff was placed on the left middle finger. A laptop was connected to the reference device, and the raw data was sampled at 1,000 Hz, and continuously recorded during the experiments using Lab Chart 8.1.9 software (AD Instruments, Sydney, Australia). During each activity, the hand with the reference device was maintained in a steady position to minimize possible noise and artifacts. Between each activity there was a pause where the reference device recording was stopped. Therefore, the finger cuff device was calibrated at the start of each activity by using a brachial cuff-based BP, which was measured on the right upper arm with a validated automated oscillometric device (Watch BP O3, Microlife Health Management Ltd., Cambridge, UK). Three readings separated by 1 min intervals were taken during the 4-minute resting period at the beginning of each of the three activities.

A 3-lead ECG was recorded continuously using Bio Amp/PowerLab (AD Instruments) during the tests and the data were exported to Lab Chart to calculate heart rate (HR).

Inclusion BP was measured on the right upper arm with the participant in the supine position before the first activity using the validated automated oscillometric device. Three consecutive measurements were taken with 1 min intervals between measurements. The first measurement was discarded, and the average of the two remaining measurements were used to calculate inclusion BP.

### 2.3 Cuffless blood pressure device

**Figure 1:**
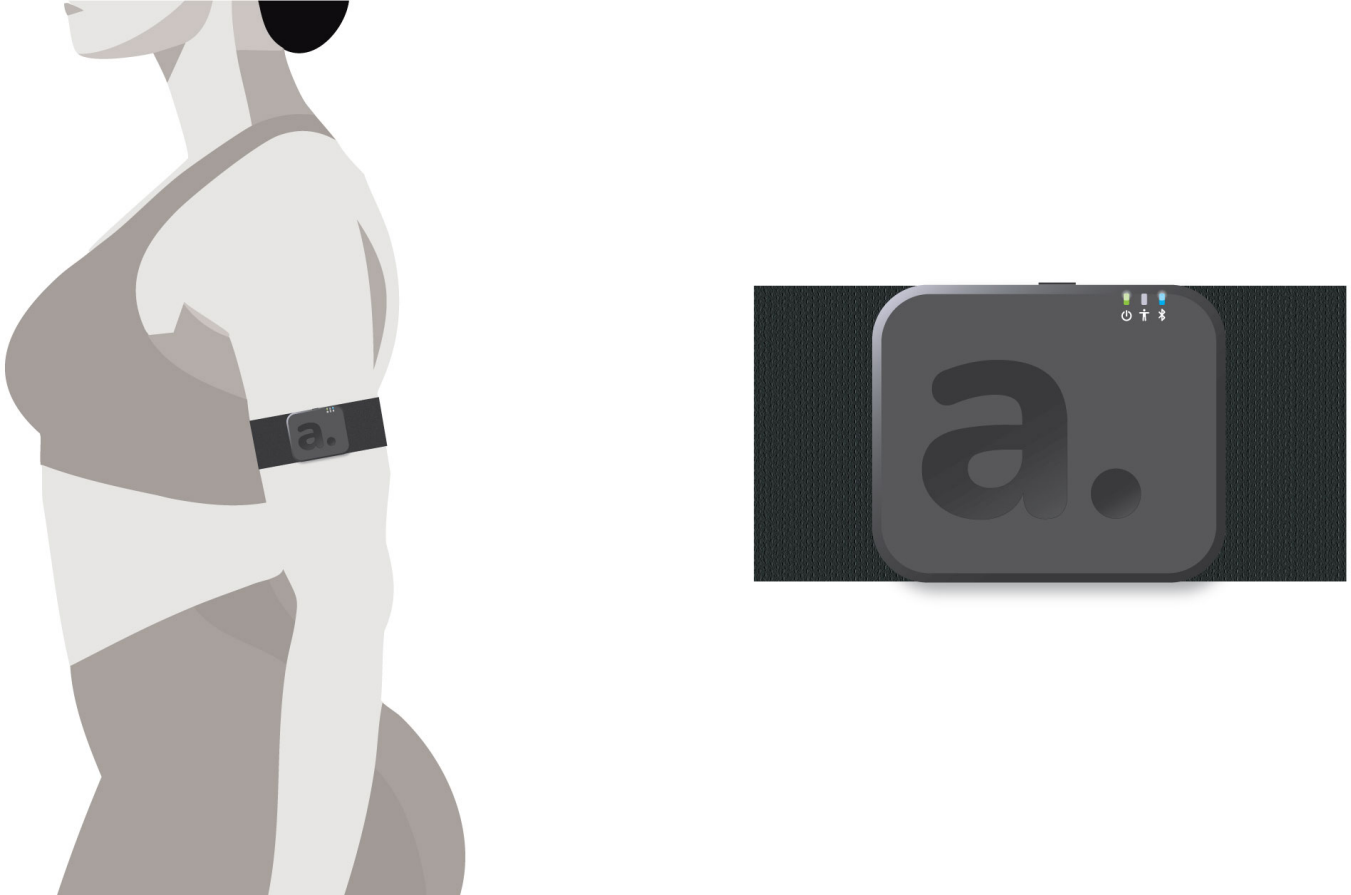
Illustration of the cuffless blood pressure device.

A prototype cuffless device, developed by Aidee Health AS (Bærum, Norway), was used in the present study (Figure 1). The device is the evolution of the technology that has been previously described in several studies (2, 36–39). It is a wearable device with a PPG and an inertial measurement unit (consisting of 3D accelerometer and gyroscope). Raw signals from the PPG sensor were sampled at 1,000 Hz while accelerometer data was sampled at 208 Hz and gyroscope data at 28 Hz. During the study the device was placed on the left upper arm.

### 2.4 Study protocol

**Figure 2:**
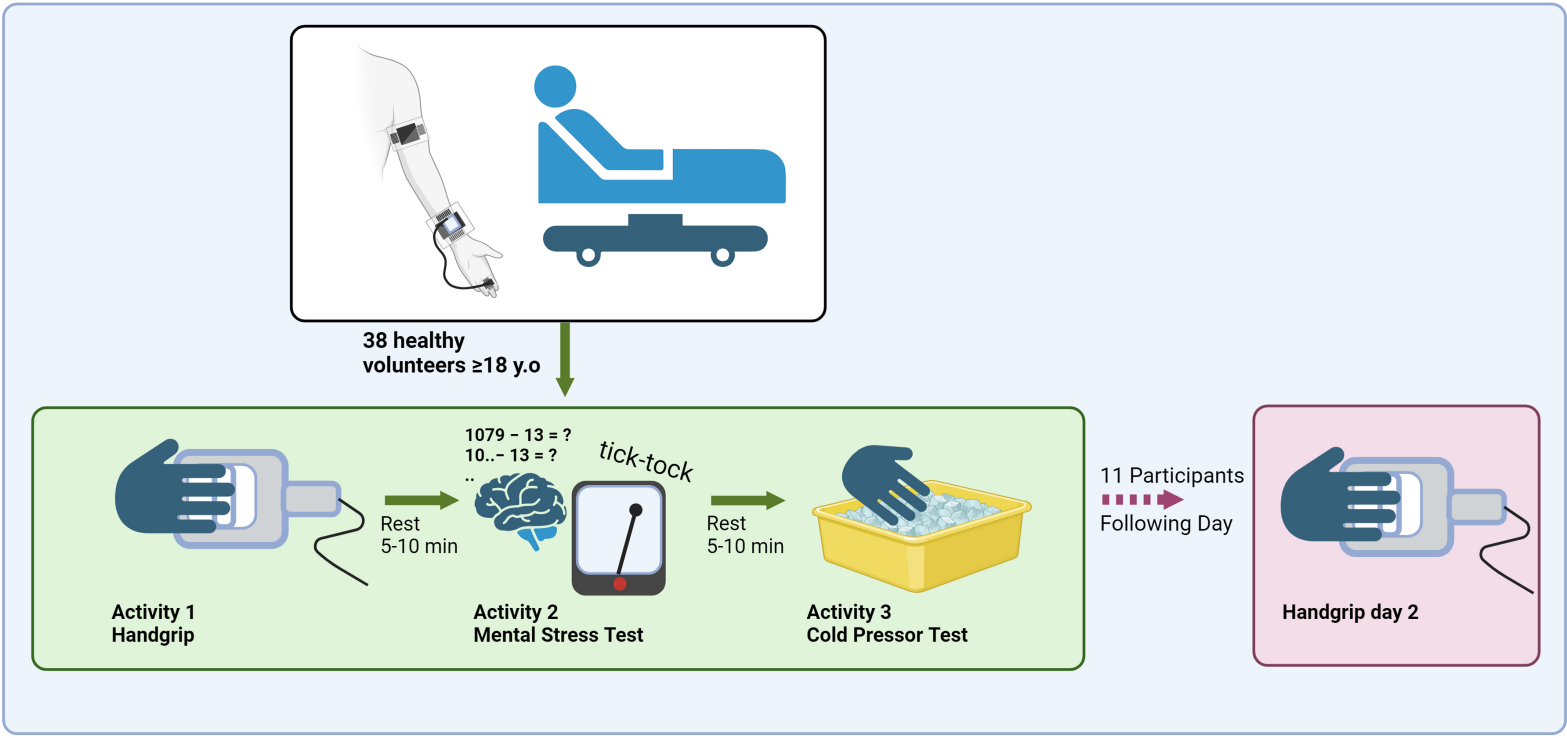
Illustration of the test protocol with activities and rest periods. Created in BioRender. Sæter, F. (2024) BioRender.com/r16m233.

The study was conducted at the Department of Vascular Surgery at Oslo University Hospital, Aker (Norway) from April to October 2023. The protocol (Figure 2) consisted of three test periods with three different activities to induce BP changes. Each period consisted of a four-minute rest followed by the test activity and 1-minute recovery. There was a longer rest of 5–10 min between each period.

Participants wore the cuffless device and the reference BP device simultaneously. The first activity, isometric handgrip, was performed by gripping the right hand around a custom-made handgrip apparatus displaying the force applied by the participant (16). Prior to the isometric handgrip, the maximal voluntary contraction (MVC) force was measured. The participants were instructed to keep 30% of MVC by looking at the display during the two minutes of isometric handgrip, avoid the Valsalva maneuver and relax all the muscles not primarily involved in contraction. This was repeated three times with two-minute pauses between each session of handgrip.

The second activity was a mental stress test where participants subtracted 13 repetitively for five minutes starting with 1,079 (17, 18). They were informed of any miscalculation in a direct and stressful manner. A metronome at a frequency of two Hz was used to distract and stress the participants.

The third activity was a cold pressor test (19–21) where the right hand of the participant was completely immersed in ice water (2–5°C) for two minutes.

Some of the participants wore the cuffless device for 24 h after the laboratory tests in order to test the stability of the cuffless BP estimations. The participants did not wear the reference device outside of the laboratory. On the following day, we repeated the isometric handgrip test with the participant wearing both the cuffless device and the reference device.

### 2.5 Data processing

Filtering and processing of the data was performed post-hoc by using Python programming language.

#### 2.5.1 Reference blood pressure

Reference BP values were calculated from the recorded raw BP waveforms and calibrated using brachial systolic BP (SBP) measurement. The brachial measurement was the mean of the last two of three measurements measured in the rest period before each activity. The raw BP waveforms were then shifted to align the peaks with the calibration measurement.

The raw waveform signals were automatically filtered to remove artefacts, such as periods of automatic calibration (AutoCal/Physiocal) and high frequency noise. Then for each cardiac cycle, defined by R-peaks in the ECG signal, the systolic, diastolic (DBP), and mean arterial pressure (MAP) were computed using maximum, minimum and time-weighted integral correspondingly. Then, all data was controlled manually for artefacts by comparing systolic and diastolic values with peaks and by reviewing actual BP waves for every subject. Finally, mean SBP, DBP and MAP were calculated per non-overlapping 15-second segments.

Participants were excluded from the statistical analyses if more than 50% of their reference data had to be removed due to artefacts or noise.

#### 2.5.2 Cuffless device

The raw PPG signals from the cuffless device were processed and filtered using proprietary algorithms. The signals were divided into cardiac cycles and averaged over non-overlapping 15-second segments. Segments with unacceptable data quality, i.e., artefacts or noise, were removed. For each 15-second segments multiple standard features from the PPG signal commonly presented in the literature (40–42) were extracted.

#### 2.5.3 Cuffless blood pressure models and calibration

**Figure 3:**
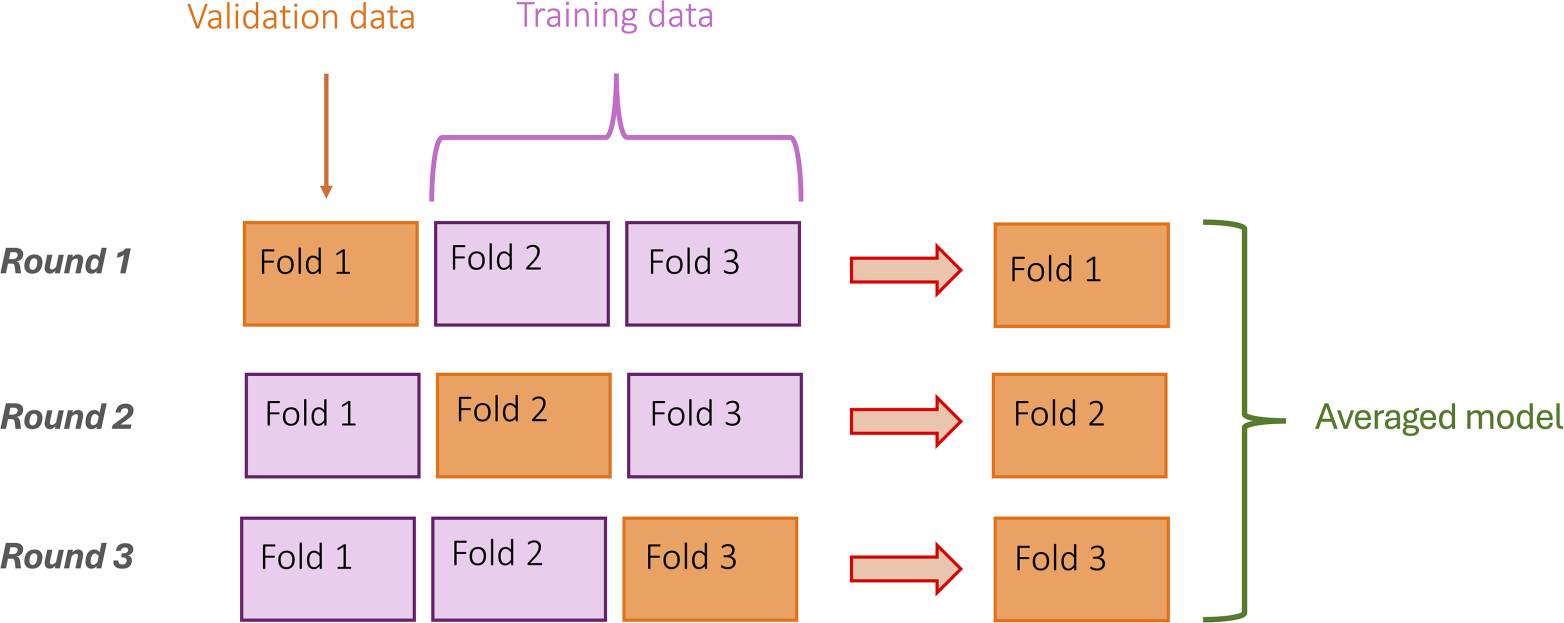
Illustration of the 3-fold cross validation procedure.

The cuffless BP models were developed from the present study cohort using 3-fold Cross-validation (43–45), a statistical method to evaluate the performance of the model in case of limited data (46). Separate models were made for each BP parameter (i.e., SBP, DBP and MAP) using the following procedure: First, the subjects were split into three subsets (“folds”), which were then used to train three different regression models for each BP parameter (Figure 3). Then a final model for each BP parameter was derived (based on averaging the three regression models). The final three models were then used to predict SBP, DBP and MAP separately.

Contrary to the reference BP, the cuffless device was only calibrated once to correct the offset between reference BP and cuffless BP. This was done during the initial rest period before the handgrip activity using the calibrated reference BP value. Participants’ demographics were not used for additional calibration.

### 2.6 Statistical analyses

Statistical analysis was performed using Stata 18.0 (Statacorp., Texas, USA). Variables were assessed for normality by visual inspection of histograms. Continuous data are presented as mean (standard deviation; SD), or median (interquartile range; IQR) if non-normally distributed. For each participant, within-subject change in BP and HR was calculated by taking the highest reported reference BP or HR measurement subtracted by the lowest reported reference BP measurement during the entire test period day 1 (handgrip, mental stress test, cold pressor test) and for each activity separately.

We chose to adopt, as closely as possible given the differences in protocol, the same statistical methodology as described in ISO 3. The ISO 3 includes three requirements for evaluating performance of cuffless devices: (1) the Accuracy criteria [Chapter 5.1 (15)], (2) the Stability criteria [Chapter 5.2 (15)] and (3) the Change criteria [Chapter 5.3 (15)]. We have compared our results against the acceptance criteria for all three tests. The acceptance criteria for the Accuracy and Stability requirements from ISO 3 is a mean difference (SD) ≤6 (10) mmHg. The acceptance criterion for the Change requirement is two-folded: averaged calculated (1) 50th percentile of error rate between the reference device and the cuffless device for the specified change evaluation interval ≤25%, and (2) 85th percentile ≤50%. To evaluate the Accuracy and Change criteria, we used the data collected from the whole test period of the first day (handgrip, mental stress test and cold pressor test). For the BP change parameters included in the Change analysis, the start/end points for BP change were kept within the same activity (either handgrip, mental stress test or cold pressor test). In the data analysis for the Stability criteria, we used data from the participants included in the 24 h test: the data collected during the first day's test period (handgrip, mental stress test and cold pressor test) and during the test period of the following day (handgrip day 2).

In addition, we evaluated the level of absolute agreement between the reference BP device and the cuffless device for SBP, DBP and MAP, during the entire first day, using Bland-Altman plots with bias and 95% limits of agreement (LoA). We acknowledge that aggregating all measurement pairs across all patients may violate the assumption of independent measurements in the Bland-Altman method (47). However, most cuffless studies have adopted this approach in their analyses (48–52).

## 3 Results

### 3.1 Participant selection, general characteristics and blood pressure distribution

**Figure 4:**
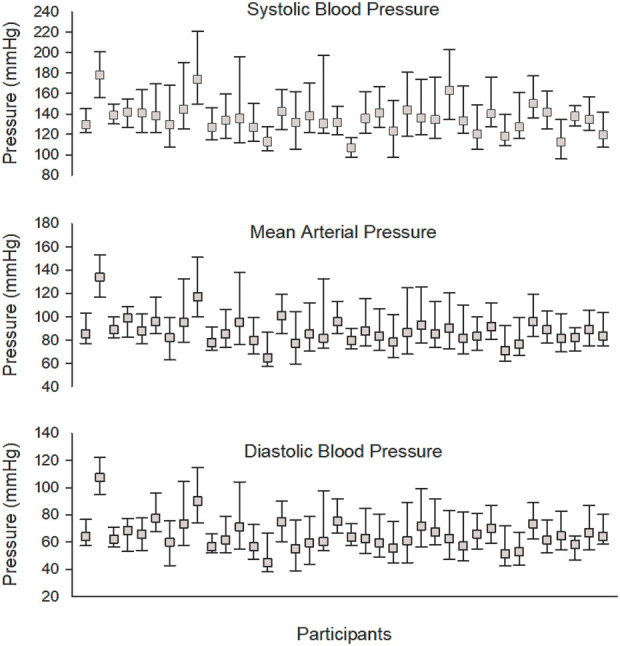
Box plot of blood pressure distribution for each individual.

A total of 67 participants were recruited, of whom 29 were excluded due to unacceptable noise in the reference BP data. Thus, 38 participants were included in the statistical analyses. General characteristics for the cohort are presented in Table 1. The BP range for each individual during the entire test protocol is presented in Figure 4. Reference BP and HR distribution during the test protocol are presented in Table 2.

**Table 1:** General characteristics of the included participants (*n* = 38).

Number (%)	38 (100)
Handgrip (day 1)	38 (100)
Mental stress test (day 1)	36 (94.7)
Cold pressor test (day 1)	33 (86.8)
24 h test (handgrip day 2)	11 (28.9)
Age, median (IQR), years Number (%)	33 (20)
• >50 years • >60 years • >70 years	5 (13.2) 2 (5.3) 0 (0)
Female sex, number (%)	22 (57.9)
Body mass index, mean (SD), kg/m^2^	23.4 (2.8)
Baseline Systolic Blood Pressure (supine position), mean (SD), mmHg	119.3 (9.4)
Baseline Diastolic Blood Pressure (supine position), mean (SD), mmHg	72.6 (7.1)
Fitzpatrick skin pigmentation, no (%)	
1	5 (13.2)
2	27 (71.1)
3	4 (10.5)
4	1 (2.6)
5	1 (2.6)
6	0 (0)
7	0 (0)

IQR, interquartile range; SD, standard deviation.

**Table 2:** Blood pressure and heart rate distribution of all individual measurements during the entire test protocol.

	Systolic blood pressure, mmHg	Diastolic blood pressure, mmHg	Mean arterial pressure, mmHg	Heart rate, beats per minute
All activities day 1				
Range, min–max	96–221	38–122	57–153	41–108
Within-subject change, median (IQR)	41.3 (26.5)	27.1 (10.7)	32.1 (13.1)	27.5 (10.1)
Handgrip (day 1)				
Range, min–max	96–221	40–122	59–153	42–96
Within-subject change, median (IQR)	25.5 (22.6)	17.1 (11.6)	21.7 (13.5)	15.8 (8.5)
Mental stress test (day 1)				
Range, min–max	106–198	38–108	57–135	45–108
Within-subject change, median (IQR)	22.6 (15.1)	13.0 (9.0)	16.2 (11.7)	21.6 (13.6)
Cold pressor test (day 1)				
Range, min–max	98–201	42–119	62–149	41–100
Within-subject change, median (IQR)	35.1 (19.0)	21.0 (12.2)	27.3 (15.0)	14.1 (8.1)
Handgrip day 2				
Range, min–max	105–196	39–104	60–138	44–98
Within-subject change, median (IQR)	33.3 (23.1)	19.1 (17.2)	23.6 (19.1)	15.7 (7.7)

IQR, interquartile range.

### 3.2 Performance of the cuffless blood pressure model

To determine the minimum number of repeated paired measurements and number of subjects, the intraclass correlation coefficient (ICC) was estimated *a priori* as outlined in ISO 3. *Post-hoc* the ICC, that was calculated from the reference data included in the analysis, was 0.2 for SBP, 0.3 for DBP and 0.2 for MAP for the Accuracy analysis, and 0.3 for SBP, 0.3 for DBP and 0.3 for MAP for the Stability analysis.

Twenty-two randomly chosen pairwise comparisons between reference and cuffless BP per subject, i.e., a total of 836 measurement pairs for each BP parameter, were used to evaluate the Accuracy criteria. Forty-four randomly chosen pairwise comparisons between reference and cuffless BP per subject, i.e., a total of 484 measurement pairs for each BP parameter, were included in the Stability analysis. A total of 3,549 measurement pairs for SBP, 3,142 for DBP and 3,510 for MAP were included in the Change analysis.

Table 3 summarizes the comparison between the cuffless device and the reference BP device, with respect to the acceptance criteria outlined in ISO 3. The mean difference (SD) was 0.3 (8.7) mmHg for SBP, 0.04 (6.6) mmHg for DBP, and 0.8 (7.9) mmHg for MAP for the Accuracy requirement. The corresponding mean differences for the Stability requirements were 1.9 (9.2), 2.9 (8.1), and 2.5 (9.5) mmHg for SBP, DBP and MAP, respectively. Thus, all BP parameters were within acceptance criteria for the Accuracy and Stability requirements (≤6.0 (10.0) mmHg).

**Table 3:** Performance of the cuffless blood pressure device in comparison to the ISO 81060-3:2022 acceptance criteria.

	Accuracy Mean Δ (SD), mmHg	Stability^a^ Mean Δ (SD), mmHg	*ISO Criteria for* *Accuracy and Stability* *Mean Δ (SD), mmHg*	Change 50th and 85th percentile, %	*ISO Criteria for* *Change 50th and* *85th percentile, %*
Systolic blood pressure	0.3 (8.7)	1.9 (9.2)^a^	≤*6.0* (*10.0)*	39, 56	≤*25*, ≤*50*
Diastolic blood pressure	0.04 (6.6)	2.9 (8.1)^a^	≤*6.0* (*10.0)*	32, 48	≤*25*, ≤*50*
Mean arterial pressure	0.8 (7.9)	2.5 (9.5)^a^	≤*6.0* (*10.0)*	33, 47	≤*25*, ≤*50*

^a^ Only 11 participants were included in the Stability analysis.

Δ, difference; SD, standard deviation.

Text and values in Italic font indicate the pass criteria of the ISO 81060-3:2022 standard.

The 50th and 85th percentile of error rate between the reference device and the cuffless device was 39% and 56% for SBP, 32% and 48% for DBP and 33% and 47% for MAP, respectively. Thus, the cuffless device achieved the acceptance criteria for the Change requirement for the 85th percentile of ≤50% error for DBP and MAP but were higher than acceptable for SBP (55% vs. ≤50%) and for all parameters for the 50th percentile of error rate (32%–39% vs. ≤25%).

**Figure 5:**
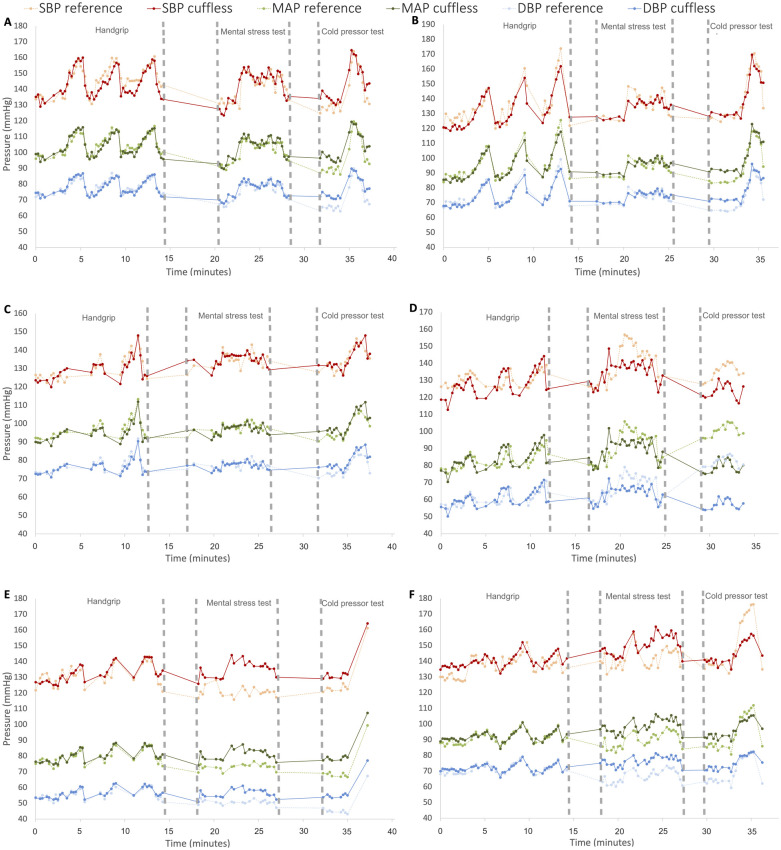
Time series plots of the results from the entire test protocol day 1 from six different participants for reference and cuffless systolic blood pressure (SBP), diastolic blood pressure (DBP) and mean arterial pressure (MAP). The *y*-axis represents blood pressure (mmHg) and *x*-axis time (minutes). The results are from three subjects with good agreement **(A**–**C)** and three subjects with mediocre agreement **(D**–**F)**.

To exemplify the results, we included time series plots from six selected participants in Figure 5. Note that because the reference device was calibrated for each activity, while the cuffless device was calibrated only once, there is a notable offset between BP readings for certain participants and activities (see Figure 5). This does not influence results for Change, but the Accuracy and Stability metrics could potentially be improved if we had not recalibrated the reference BP before each activity.

**Figure 6:**
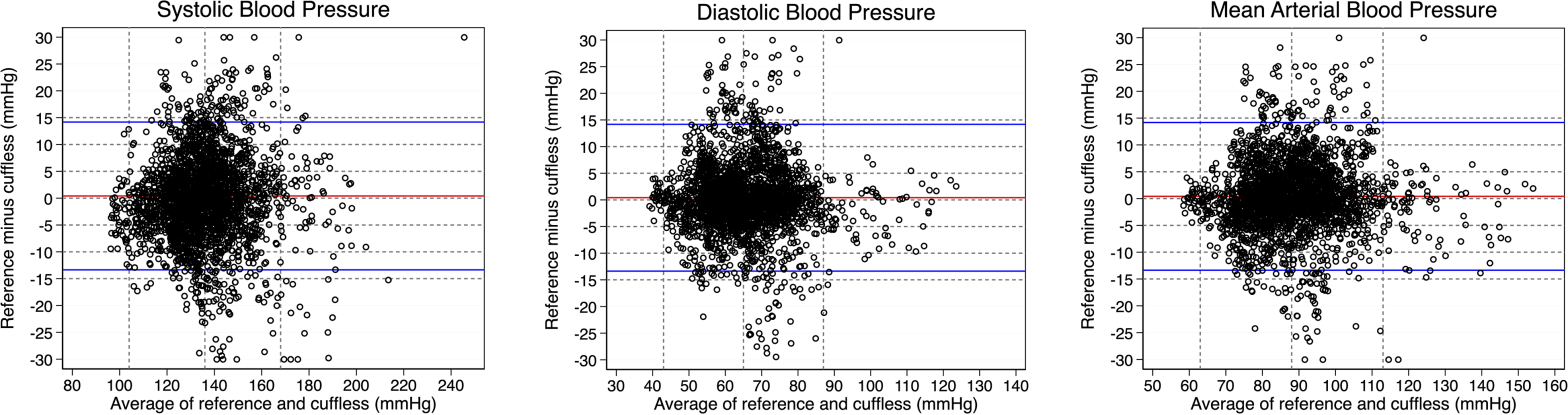
Bland-Altman plots for individual blood pressure (BP) readings for each BP parameter day 1 of the test protocol. Mean of BP values from the reference device and cuffless device (*x*-axis) plotted against the difference between reference and cuffless BP values (*y*-axis). Horizontal red lines indicate bias and horizontal blue lines indicate upper and lower 95% limits of agreement. Vertical, dotted lines represent mean (±2 SD). Outliers, defined as BP differences above 30 mmHg and below −30 mmHg (53), are plotted at point 30 mmHg and −30 mmHg, respectively.

The degree of agreement between the cuffless device and reference device during the entire test protocol day 1 is presented with Bland Altman plots with bias and 95% LoA (Figure 6). Bias [95% LoA] was close to zero for all BP parameters over the entire test period day 1 (all activities); 0.24 mmHg [−8.7, 9.2 mmHg], 0.63 mmHg [−7.3, 8.5 mmHg] and 0.77 mmHg [−7.2, 8.7 mmHg] for SBP, DBP and MAP, respectively.

## 4 Discussion

The present study aimed to evaluate the ability of a prototype, PPG-based cuffless device, placed on the upper arm, to track BP during three well-known activities to induce BP changes using different physiological mechanisms. The results demonstrated that the cuffless device estimated BP with satisfactory accuracy compared to a non-invasive, continuous reference BP monitor, in 38 healthy adults in an experimental laboratory set up consisting of isometric handgrip (16), mental stress test (17, 18) and cold pressor test (19–21). The cuffless device showed promising results in achieving the acceptance criteria from the ISO 81060-3:2022 standard (ISO 3) (15). To the best of our knowledge, this is the first study to present results according to the full statistical methodology outlined in the ISO 3, which is the first standard addressing the validation of cuffless devices.

The cuffless device fulfilled the acceptance criteria (≤6 (10) mmHg) for the Accuracy and Stability requirements from ISO 3. A particular strength of our study is that these metrics were calculated using the whole measurement period, including the periods with the induced BP change (unstable periods) which is not required by ISO 3. Even though the present study is not a validation study, we adopted as closely as possible (given our different protocol), the metrics and statistical methodology from ISO 3, which addresses mentioned issues with evaluating performance of cuffless devices and represents state-of-the-art benchmark for the present and similar studies.

A few other cuffless devices have demonstrated the ability to accurately estimate BP in individuals at rest during stable conditions. A study evaluating a PPG-based cuffless device, worn as a bracelet (Aktiia), compared to auscultation in 91 adults (6) demonstrated accurate BP predictions in the seated, supine and standing position with a mean difference (SD) for SBP of 0.5 (7.8) mmHg in the sitting position, −2.4 (10.1) mmHg in the supine, and −0.6 (12.5) mmHg in the standing position. Differences for DBP readings were 0.4 (6.9) mmHg, −1.9 (7.7) mmHg, and −4.9 (9.1) mmHg respectively. Accuracy of the same device (Aktiia) was compared to auscultation in 35 elderly individuals in the seated, supine and standing position (8) and demonstrated similar results. Another study evaluating a cuffless device that is based on pulse transit time (Somnotouch-NIBP) demonstrated similar results in 33 subjects in the seated position (5). The BP estimations of a cuffless, wrist-worn or skin attached device (BioBeat), that uses pulse wave analysis of the PPG signal in combination with pulse wave transit times, was compared to the measurements of a standard sphygmomanometer device in 1,057 subjects in the seated position (7). In this study the BioBeat device was found similar to the sphygmomanometer device with high agreement and reliability levels. However, none of these studies have shown that cuffless devices can accurately track substantial physiological BP changes. This is an important aspect, as cuffless devices only track changes in BP relative to the calibration value. Thus, in a stable, resting condition where BP variations are small, a device would seem to track BP accurately even though this might not be true under larger BP changes.

Unlike most studies on cuffless devices, which predominantly focus on accuracy assessment during resting conditions, we evaluated performance of the cuffless device during large BP changes induced by three different physiological mechanisms, i.e., isometric handgrip, mental stress test, and cold pressor test. The effects of isometric exercise on the cardiovascular system were first described by Lindhard in 1920 (54). Since then, it has been shown that isometric exercise causes a concurrent increase in both SBP and DBP (36, 55–57). The BP response to mental stress is characterized by a predominant elevation in SBP, reflecting increased cardiac output driven by an increase in both stroke volume and HR, while DBP may remain relatively stable or show a modest increase (58, 59). Cold induced pain typically results in a rapid and consistent BP elevation during the stimulus due to an immediate sympathetic surge, primarily in SBP, while DBP may also rise (21, 60, 61). Despite using these different mechanisms to induce BP changes, we still demonstrated high agreement in the ability of the cuffless BP device to track SBP, DBP and MAP.

Furthermore, the cuffless device showed promising results in meeting the acceptance criteria for the Change requirement of ISO 3 (50th percentile ≤25% and 85th percentile ≤50%). The 85th percentile of error rate for MAP and DBP was within the acceptance criteria but was higher than the limit for SBP for the 85th percentile and for all BP parameters for the 50th percentile. Only one comparative study has presented results partially according to the statistical methodology outlined in ISO 3, i.e., Khayat et al. recently evaluated a wearable sensor against intra-arterial BP measurements for the Change criteria in 27 patients undergoing surgery, achieving a 50th percentile and 85th percentile of error rate of 23.8% and 42%, respectively (62), meeting the ISO 3 Change criteria (≤25% and ≤50% error for the 50th and 85th percentiles respectively). Even though the cuffless device, tested in the present study, only partially fulfilled the acceptance criteria for the Change requirement, we argue that the results are promising towards meeting the criteria in a future validation study for several reasons. First, ISO 3 only requires a limited increase (15 mmHg in SBP, 10 mmHg in DBP and 12 mmHg in MAP) for the BP change included in the Change analysis, and it does not require comparison of measurements obtained during this period of change where BP is unstable. In the present study, we induced substantial BP changes in our participants, and all available pairs were included in the Change analysis. During some activity periods BP was changing extremely fast. This might have introduced a higher uncertainty in BP measurements from both the reference and cuffless device. Thus, our results may have been better had we excluded periods with unstable BP. However, we decided not to do this to clearly illustrate the ability of the technology to track changes in challenging conditions as well. Second, ISO 3 only requires a certain BP change but does not specify how this BP change shall be achieved. Alterations in BP can be induced by different mechanisms and stimuli, and the hemodynamic responses to these can vary in terms of magnitude and duration. In the present study, we used 3 different interventions to induce BP changes through different physiological mechanisms, instead of just using one single exercise that is typically performed (36, 37, 63). Third, the activities used to induce BP changes were equally weighted in our Change calculations. The cold pressor test, where the right hand of the participant was completely immersed in ice water, could in some individuals have led to a significant peripheral vasoconstriction in the extremity contralateral to the cold immersion (64) and probably introduced a higher uncertainty in the BP measurements conducted by the reference device (64, 65). We believe that these factors may explain why we did not fulfill all acceptance criteria outlined in ISO 3 in the present study and argue that the cuffless device showed promising results in meeting the Change requirements in a future validation study.

## 5 Deviances from the ISO 81060-3:2022 protocol

Even though we used methodology from ISO 81060-3:2022 to evaluate our results, it is important to note that the protocol used in the present study differs from the protocol described in ISO 3.

Most importantly, we used a different reference method. The ISO 3 protocol requires an intra-arterial BP reference. This involves cannulation of a peripheral artery, most commonly the radial artery, with a catheter. In the present study, the Human NIBP and Nexfin, which deliver continuous BP readings via a non-invasive dual finger cuff system, were used as reference BP devices. Both instruments use the volume-clamp methodology to assess arterial pressure in the finger and by that calculate BP (66). The accuracy of the Human NIBP Nano is according to the manufacturer ± 1% of the full range (max. 3 mmHg) (65). Nexfin has been compared to intra-arterial measurements in several studies, demonstrating bias (SD) ranging from −1.2 (6.5) mmHg to −4.6 (6.5) mmHg (27–30). The parent technology, Finapres (FMS Finapres, Medical systems BV, Amsterdam), has been demonstrated to be accurate when compared to intra-arterial pressure with only minor discrepancies (17, 34) and has been validated for use in research (31, 32). Finapres has proven to be reliable in monitoring BP during dynamic changes (67, 68). However, a few studies have shown these and comparable devices to be less accurate than intra-arterial BP measurements (69), especially for SBP (33, 70), and they are not recommended for hemodynamic monitoring in critically ill patients where sudden hypotension may occur (71). Nevertheless, the finger cuff devices are commonly accepted as reliable for non-invasive BP measurements in non-critically ill patients (17, 34, 35). While the use of intra-arterial measurements provides enhanced accuracy, it requires an invasive procedure, thereby also raising ethical, practical, and financial considerations. Therefore, for our healthy population the volume-clamp device was considered adequate and appropriate.

Second, the total test period for each subject did not fulfill the requirement for the Stability requirement of ISO 3. The standard requires measurements during the first 5 h and again after 24 h for devices intended for 24 h monitoring. This was not feasible for the present study.

Third, the number of test subjects was lower than required for the Stability requirement. For the given ICC in our dataset, ISO 3 requires at least 30 test subjects. We included more than an adequate number of test subjects (*n* = 38) for the Accuracy and Change analysis. However, only 11 individuals completed the 24 h measurement providing data for the Stability analysis.

Fourth, the subject characteristics, including the distribution of BP, did not fulfil the criteria of ISO 3. Our cohort was relatively young, i.e., only 13% >50 years (requirement >40%), 5% >60 years (requirement >25%) and none >70 years (requirement >10%).

## 6 Strengths and limitations

A strength of the present study is that we evaluated performance of the cuffless device during relatively large BP changes. This aspect is essential, given that cuffless devices estimate BP changes relative to a calibration value. Consequently, under stable, resting settings where BP fluctuations are minimal, a device may appear to track BP accurately. However, this perceived accuracy may not hold true when there are larger variations in BP. Furthermore, we used three well-known activities to induce BP changes via different physiological mechanisms, i.e., isometric handgrip, mental stress test, and cold pressor test.

Another strength is that we assessed performance of the cuffless device in accordance with the statistical requirements from the ISO 81060-3:2022 standard. This standard provides a framework and statistics that enables direct comparisons between different continuous, cuffless devices during both stable and unstable conditions. Even though the present study is not a validation study, using these metrics is particularly meaningful for future comparisons with other devices. Additionally, we have highlighted our results along with important deviances from the ISO 81060-3:2022 protocol, which we believe are noteworthy for future investigations and device comparisons.

However, the present study has several limitations. First, the study was conducted under highly controlled laboratory conditions. Consequently, the results may not be directly applicable to real-life settings. Second, most of our participants had light skin color (Fitzpatrick 1–3). Third, we only included healthy individuals. Cuffless BP devices make presumptions about the arterial pulse wave in their BP prediction models. Thus, the BP prediction models might not be generalized to individuals with chronic diseases, such as peripheral artery disease or cardiovascular diseases, pregnant women, individuals with obesity, darker skin tones and/or tattooed skin (14). The aim of the present study was to induce relatively large BP changes. Thus, for ethical reasons we chose to exclude candidates with comorbidities, such as hypertension and cardiovascular diseases. Further research is necessary to determine device performance in these sub-populations, which are currently under-represented in clinical trials.

## 7 Conclusions

The present study demonstrated the ability of a prototype, photoplethysmography-based cuffless device, placed on the upper arm, to track large BP changes induced by different physiological mechanisms. The cuffless device showed promising results in achieving the acceptance criteria for the Accuracy, Stability and Change requirements from the ISO 81060-3:2022 standard in healthy adults. However, it is important to note, that we used a different reference BP device and did not fulfill the requirement for participant characteristics. Furthermore, the subject number and study protocol time was not in accordance with the standard for the Stability requirement.

The results of the present study are optimistic towards the clinical use of cuffless devices in BP monitoring in healthy adults. However, further research and validation is needed before the technology can be implemented in health care.

## Data Availability

BP predictions from the cuffless BP model and the reference measurements can be made available upon a reasonable request. Raw signals and data regarding model development may not be disclosed.

## Supplementary material

Supplementary Figure S7 Histogram of all reference blood pressure readings (*n* = 836) included in the ISO Accuracy analysis for each blood pressure parameter.

Supplementary Figure S8 Scatter plot of all reference blood pressure readings (*n* = 836) included in the ISO Accuracy analysis for each blood pressure parameter. The *y*-axis represents included reference blood pressure (mmHg), *x*-axis time (minutes) from calibration of the cuffless device.

Supplementary Figure S9 Histogram of all reference blood pressure readings (*n* = 484) included in the ISO Stability analysis for each blood pressure parameter.

Supplementary Figure S10 Histogram of all reference blood pressure readings (*n* = 7,098 for systolic, *n* = 6,284 for diastolic and *n* = 7,020 for mean arterial pressure) included in the ISO Change analysis for each blood pressure parameter.

Supplementary Figure S11 Histogram of all reference blood pressure change parameters (*n* = 3,549 for systolic, *n* = 3,142 for diastolic and *n* = 3,510 for mean arterial pressure) included in the ISO Change analysis for each blood pressure parameter. The reference blood pressure change parameter was calculated by taking the reported reference blood pressure measurement at the end of the blood pressure change minus the reported reference blood pressure measurement at the start of the blood pressure change.
