# Supplementary figures and images for: A prototype photoplethysmography-based cuffless device shows promising results in tracking changes in blood pressure
Source: Front Med Technol. 2024 Oct 21;6:1464473. doi: 10.3389/fmedt.2024.1464473 (PMC11532190; doi:10.3389/fmedt.2024.1464473)

Number of individual readings

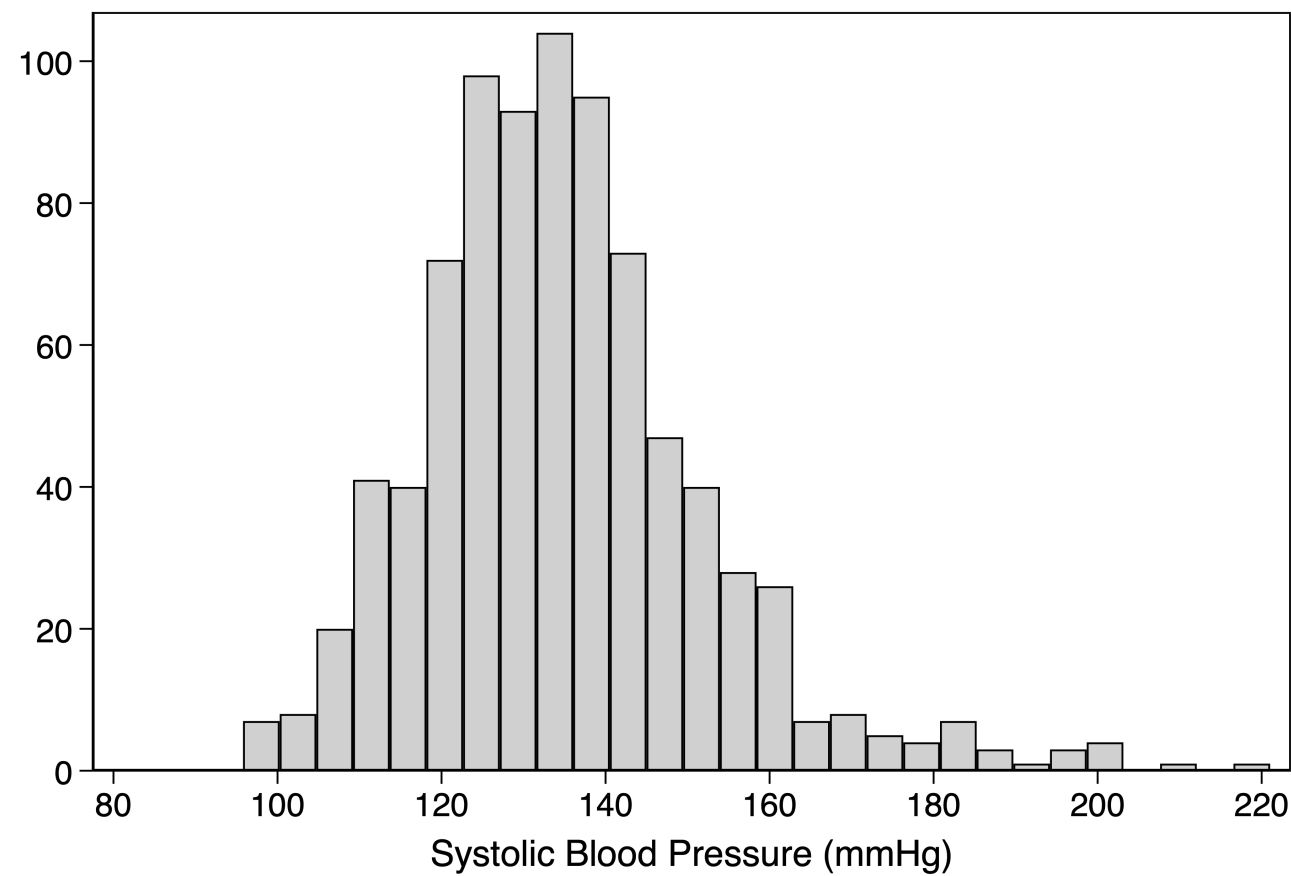

Number of individual readings

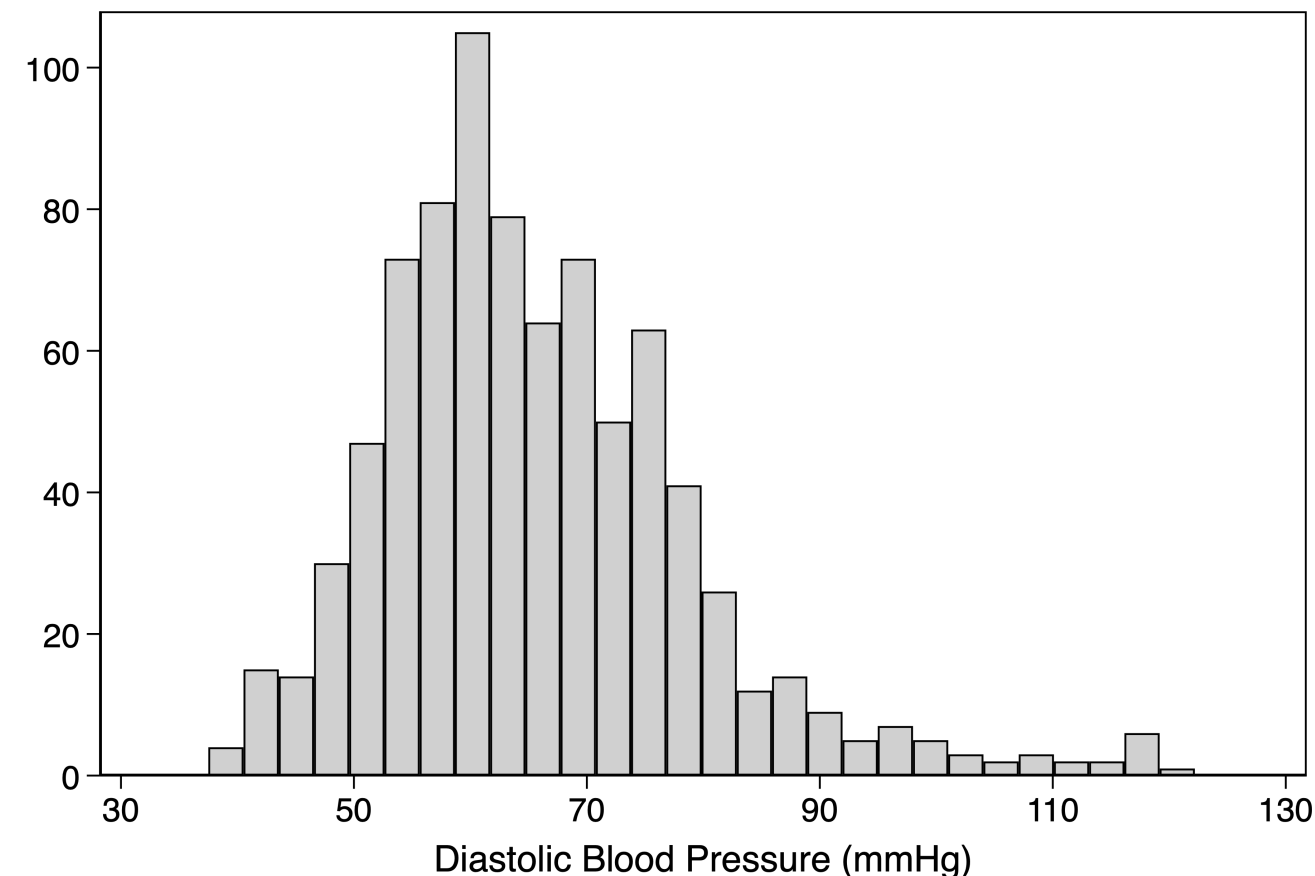

Number of individual readings

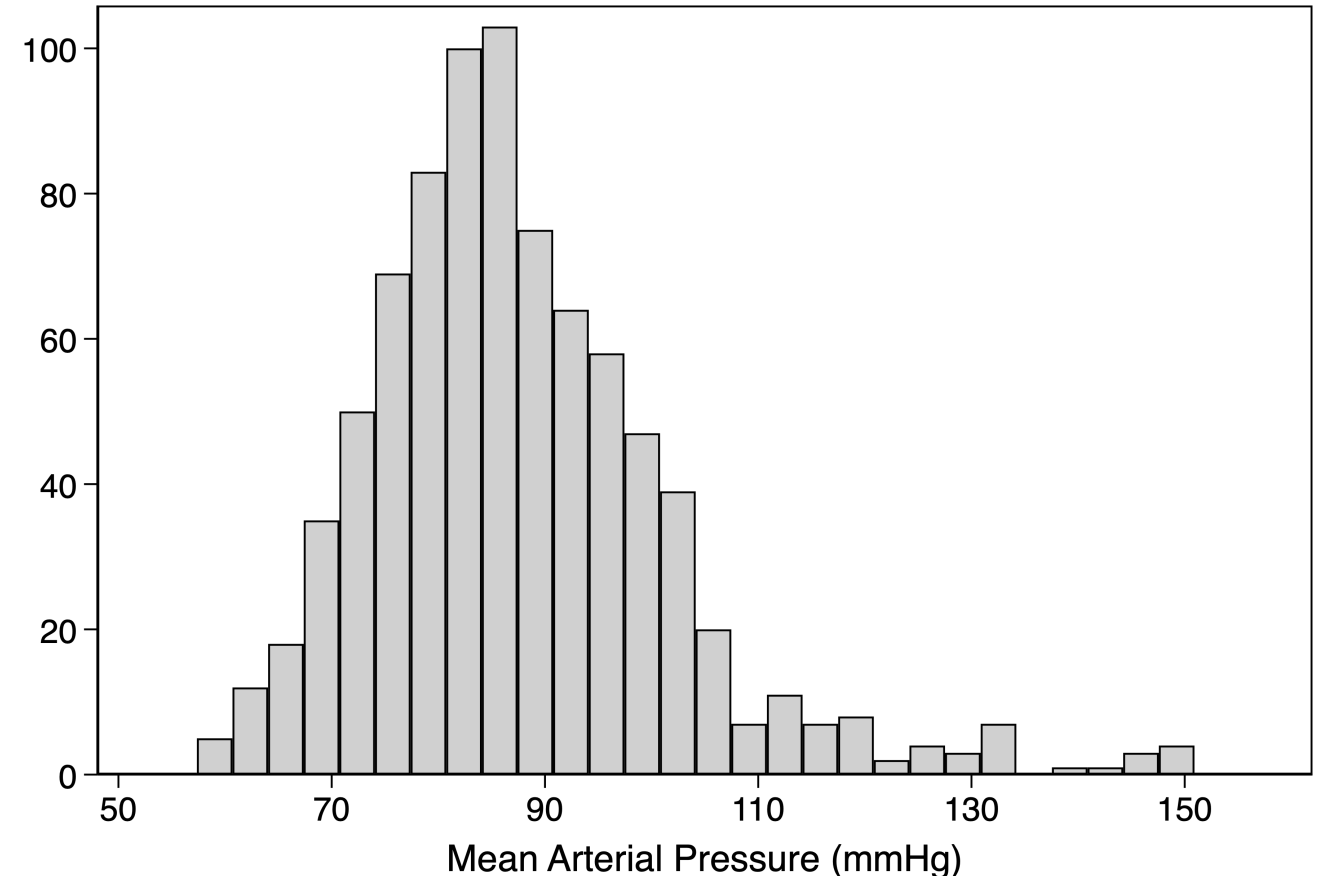

Supplement: Supplementary Figure S7 — Histogram of all reference blood pressure readings (n = 836) included in the ISO Accuracy analysis for each blood pressure parameter. [file Image7.pdf]

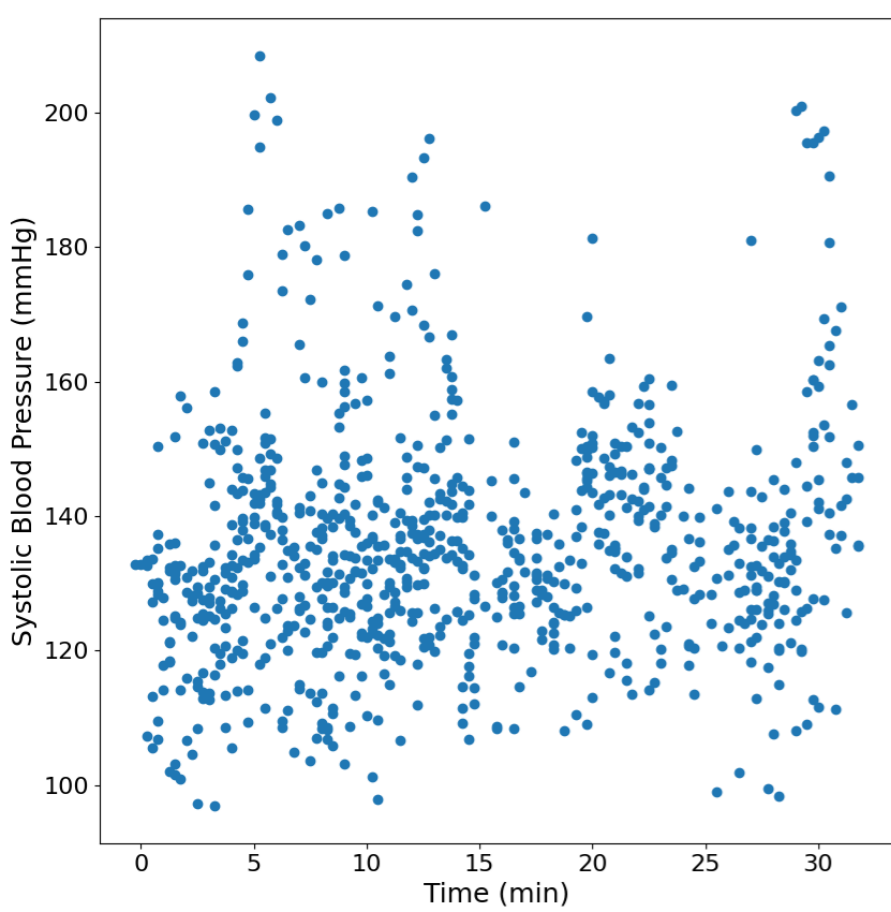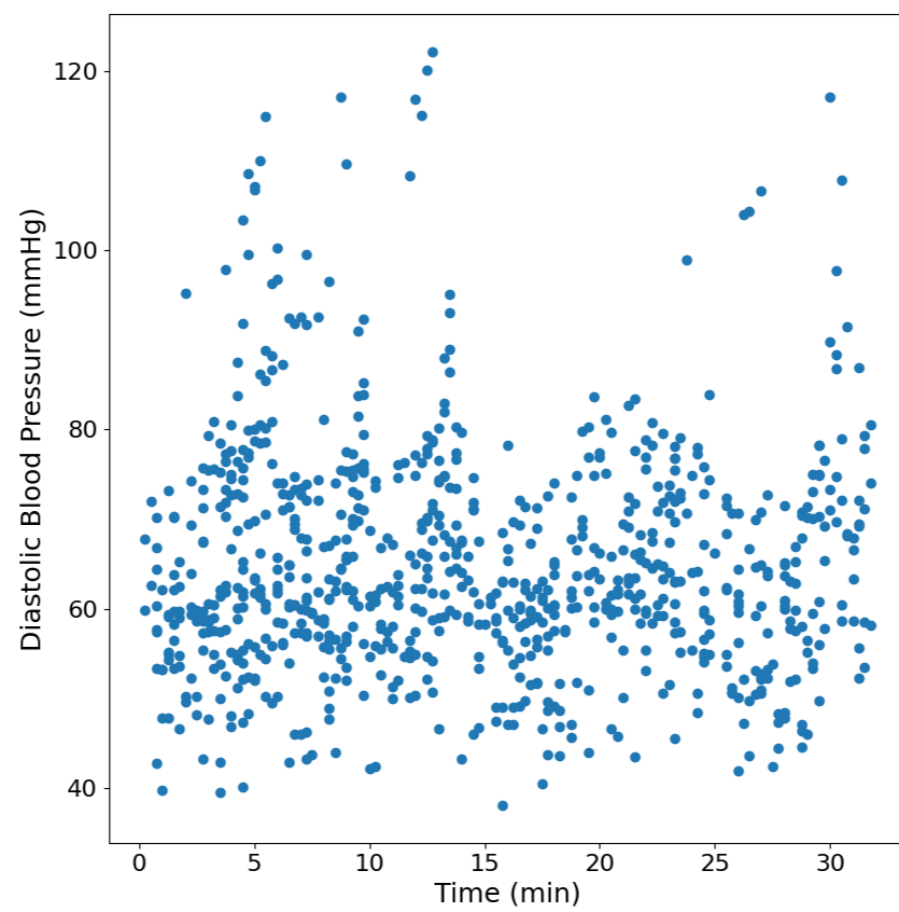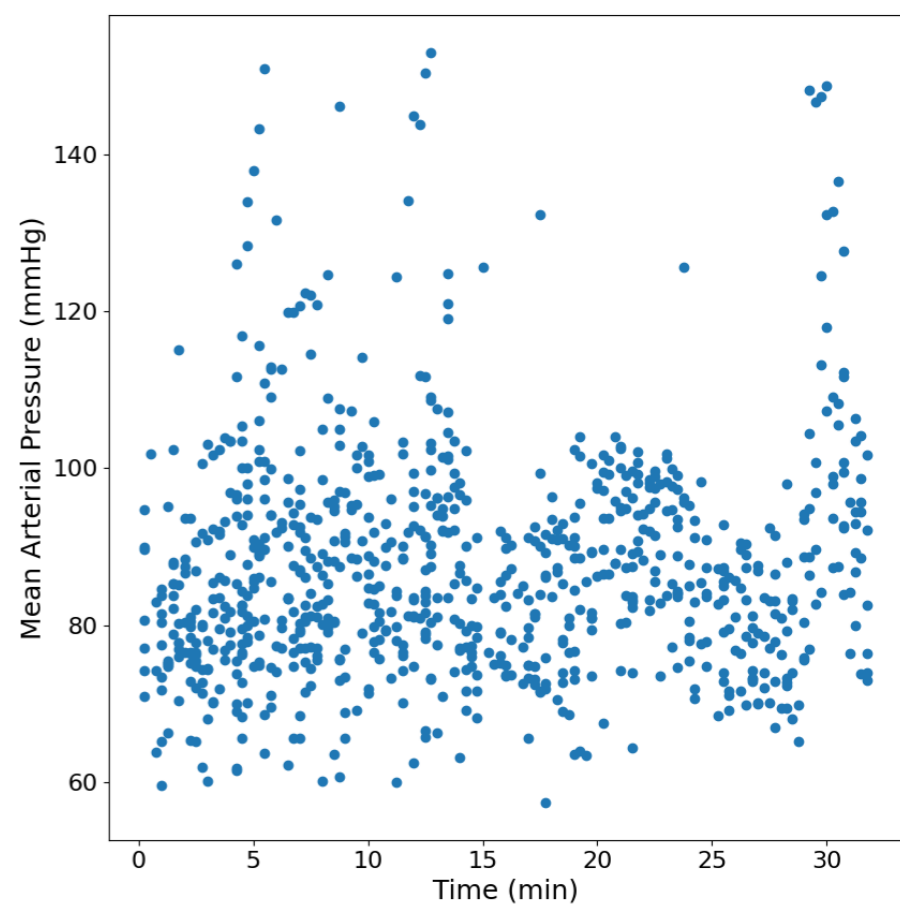

Supplement: Supplementary Figure S8 — Scatter plot of all reference blood pressure readings (n = 836) included in the ISO Accuracy analysis for each blood pressure parameter. The y-axis represents included reference blood pressure (mmHg), x-axis time (minutes) from calibration of the cuffless device. [file Image8.pdf]

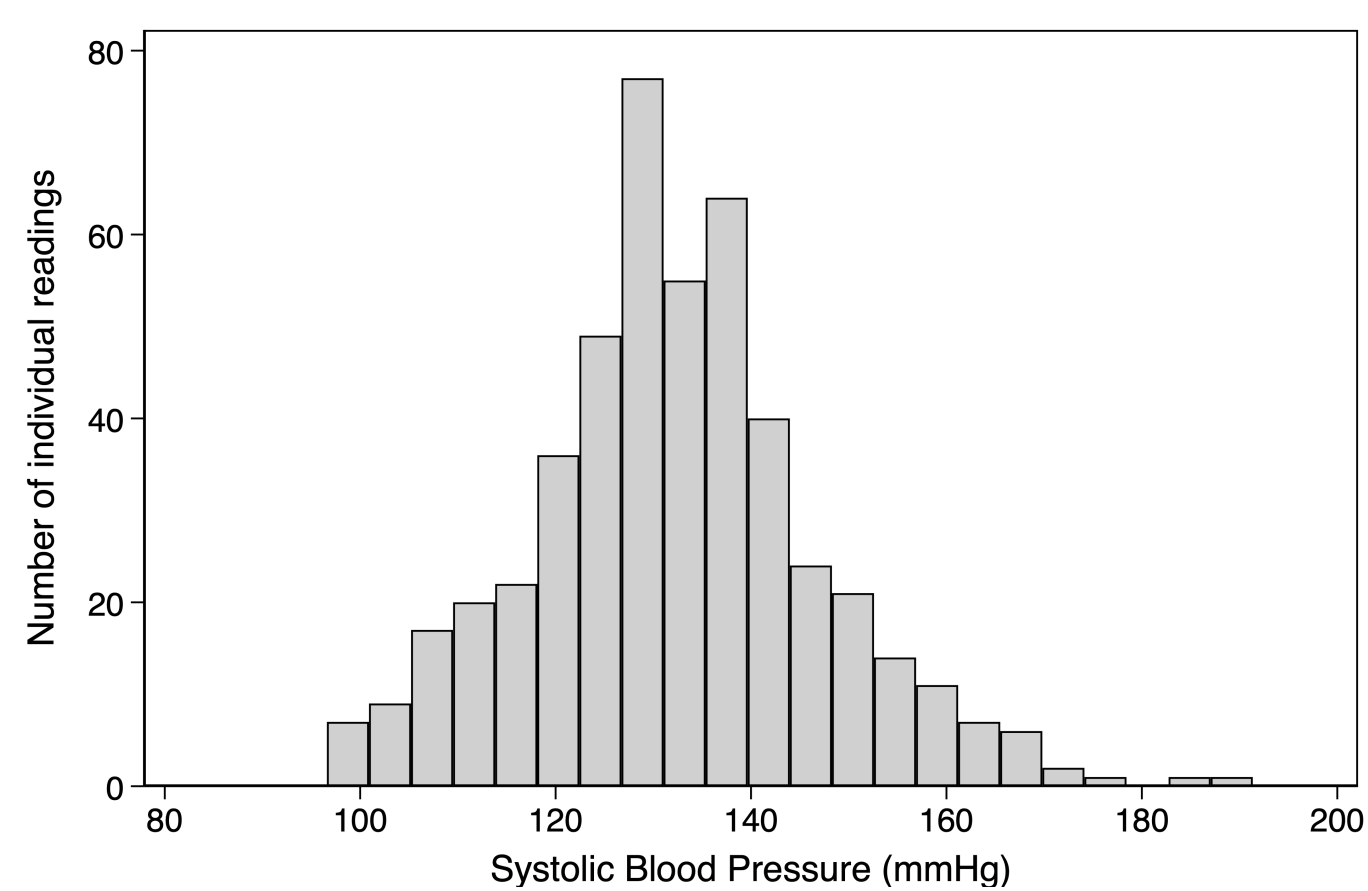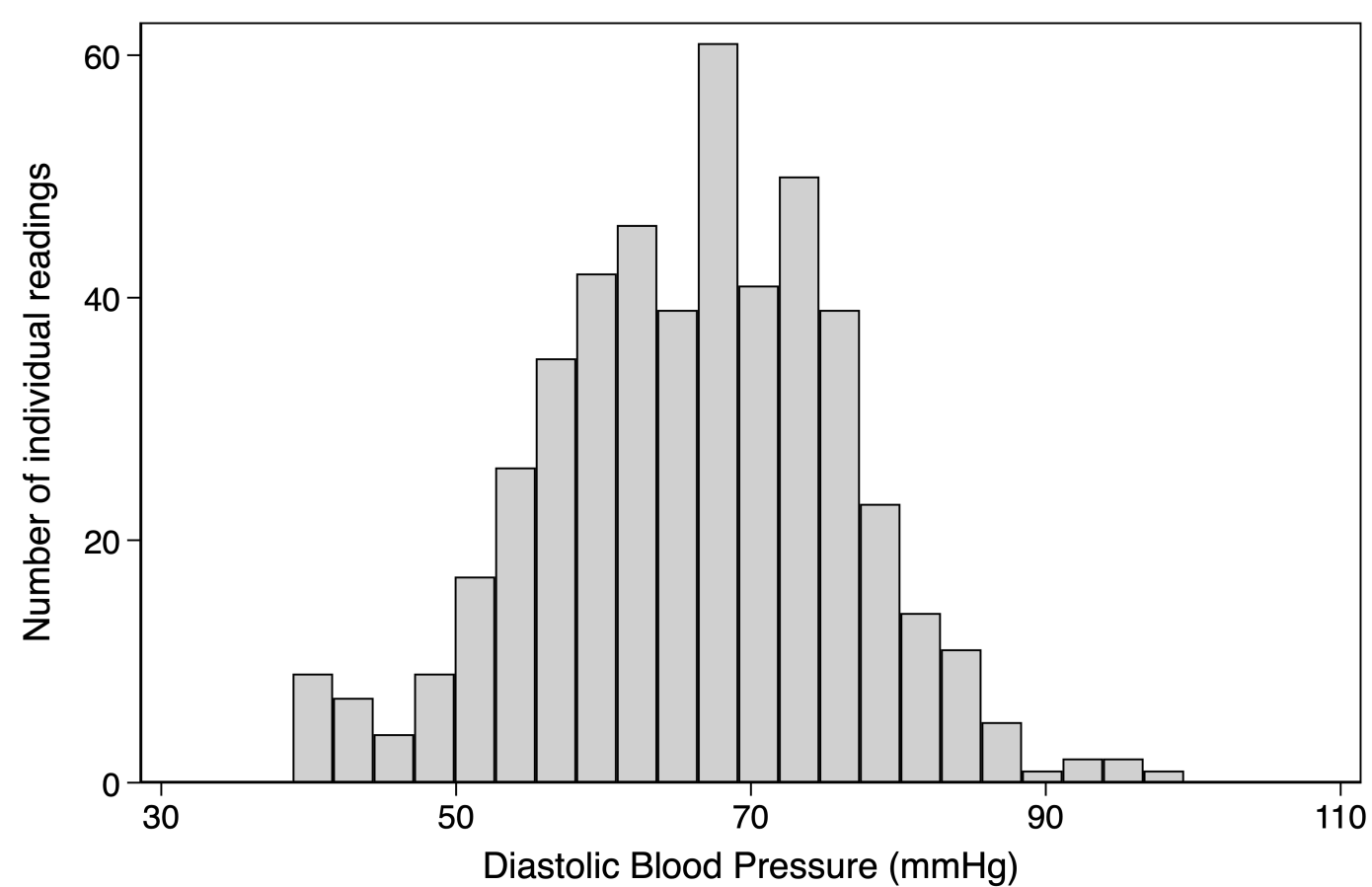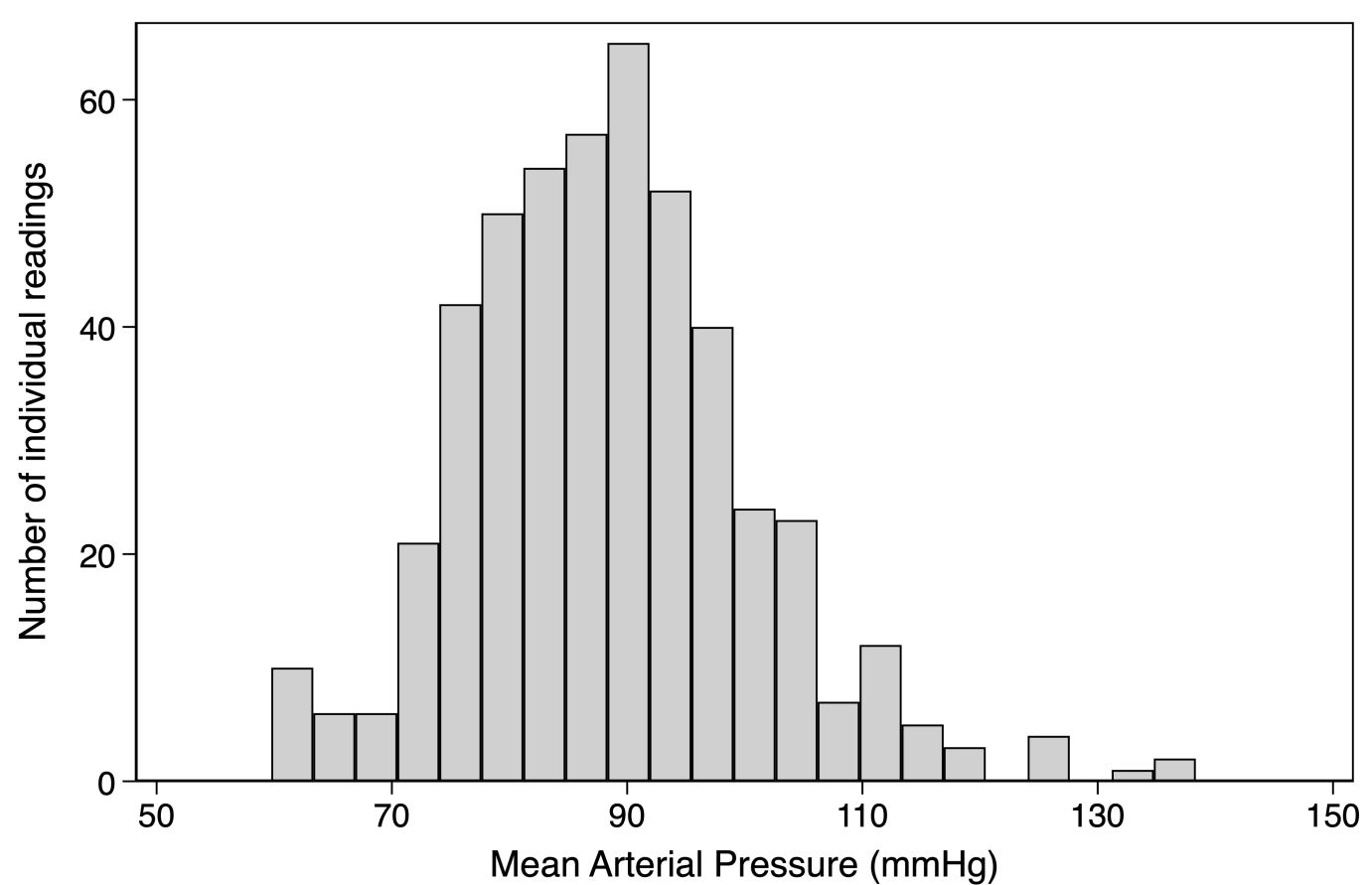

Supplement: Supplementary Figure S9 — Histogram of all reference blood pressure readings (n = 484) included in the ISO Stability analysis for each blood pressure parameter. [file Image9.pdf]

Number of individual readings

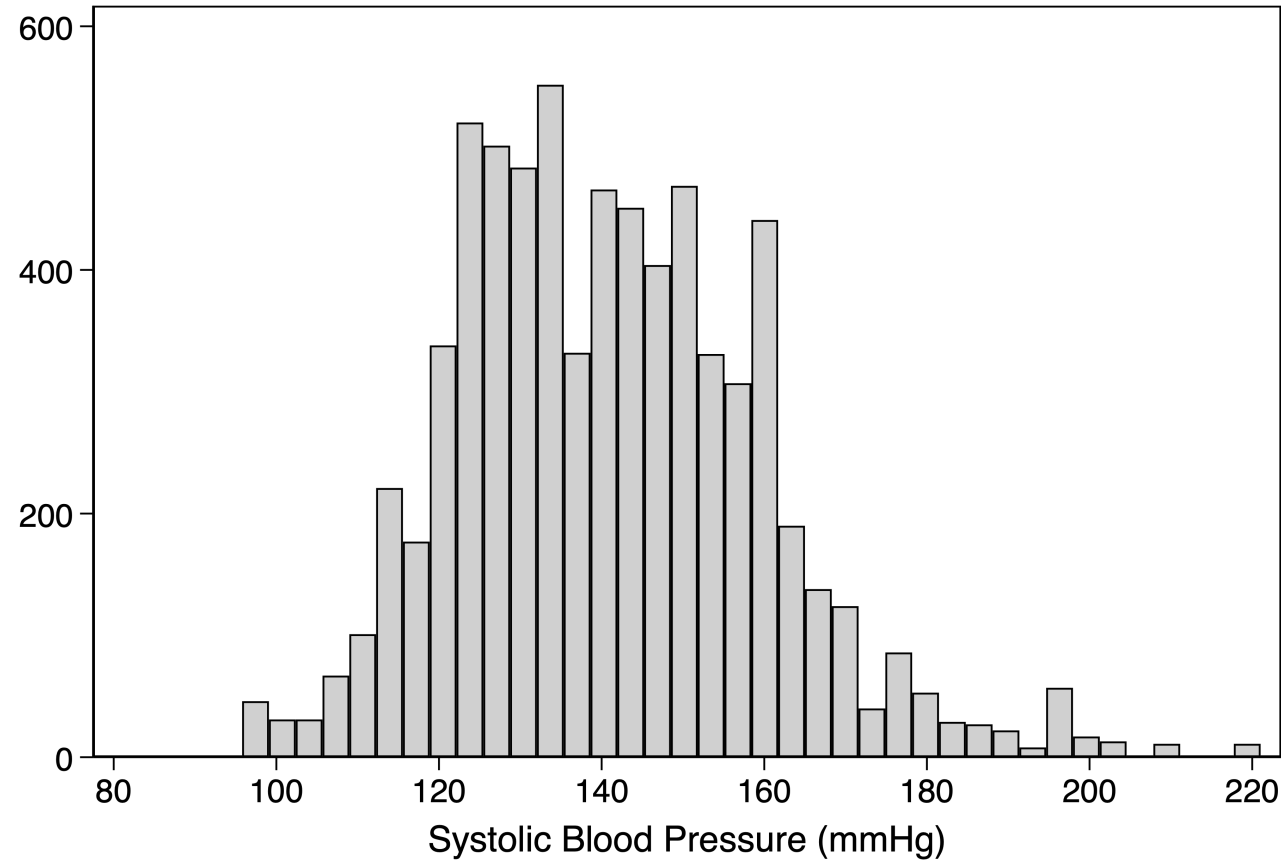

Number of individual readings

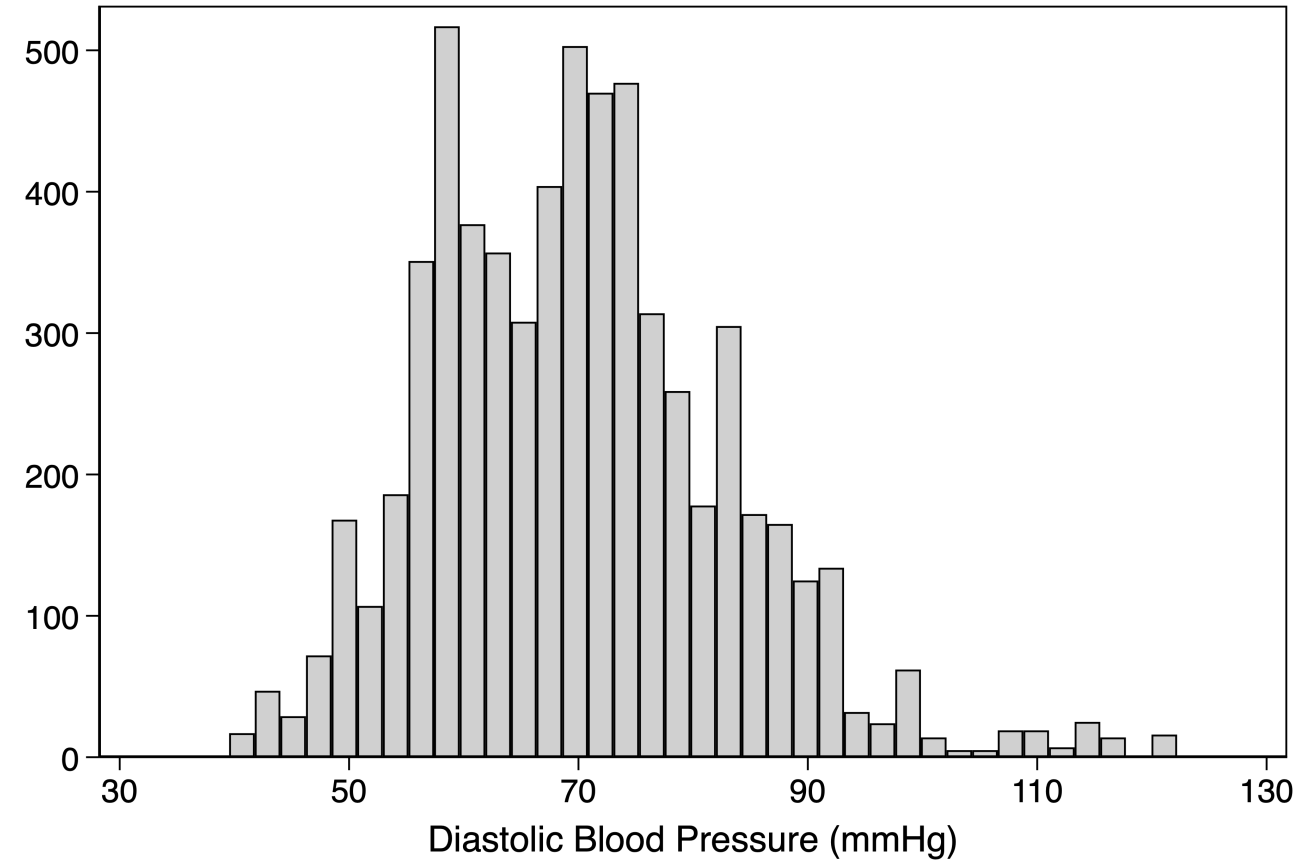

Number of individual readings

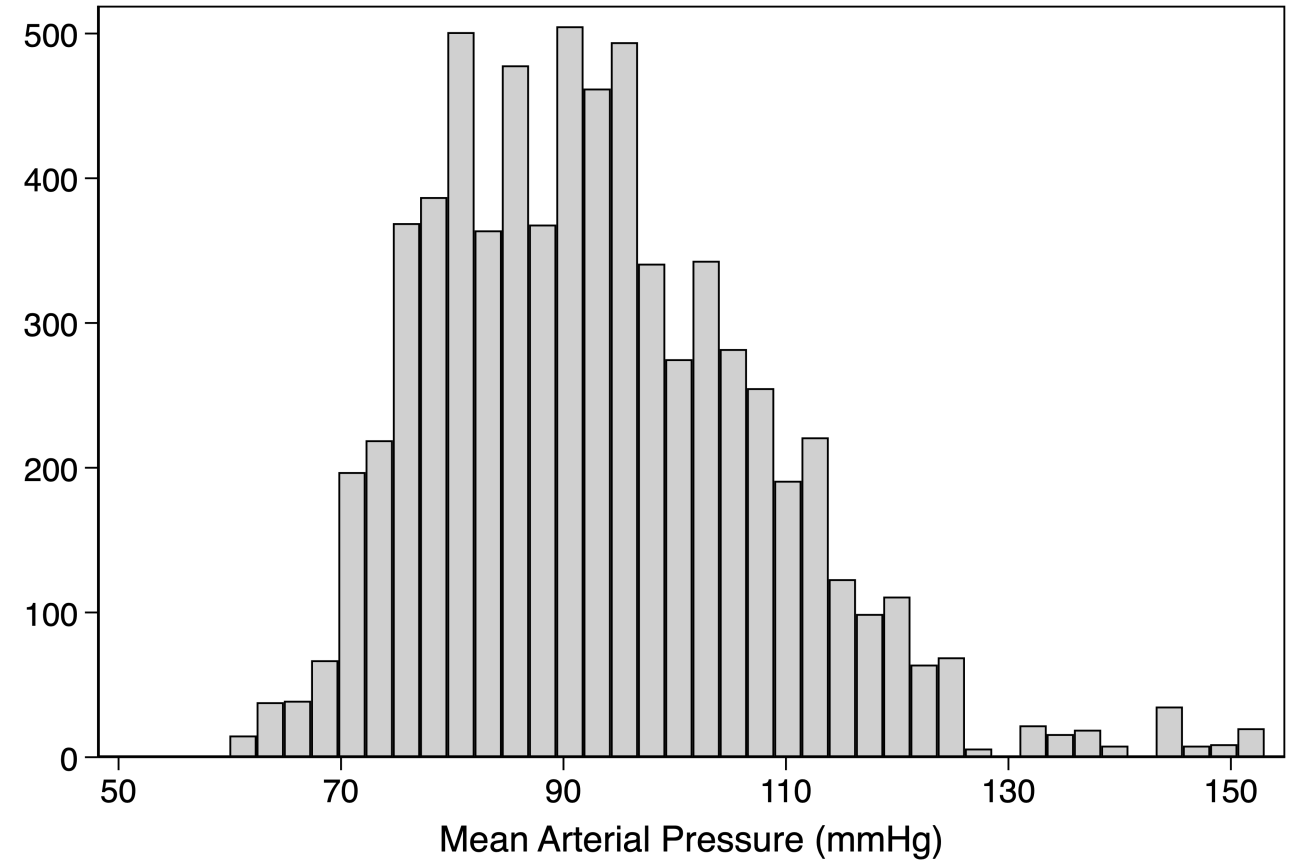

Supplement: Supplementary Figure S10 — Histogram of all reference blood pressure readings (n = 7,098 for systolic, n = 6,284 for diastolic and n = 7,020 for mean arterial pressure) included in the ISO Change analysis for each blood pressure parameter. [file Image10.pdf]

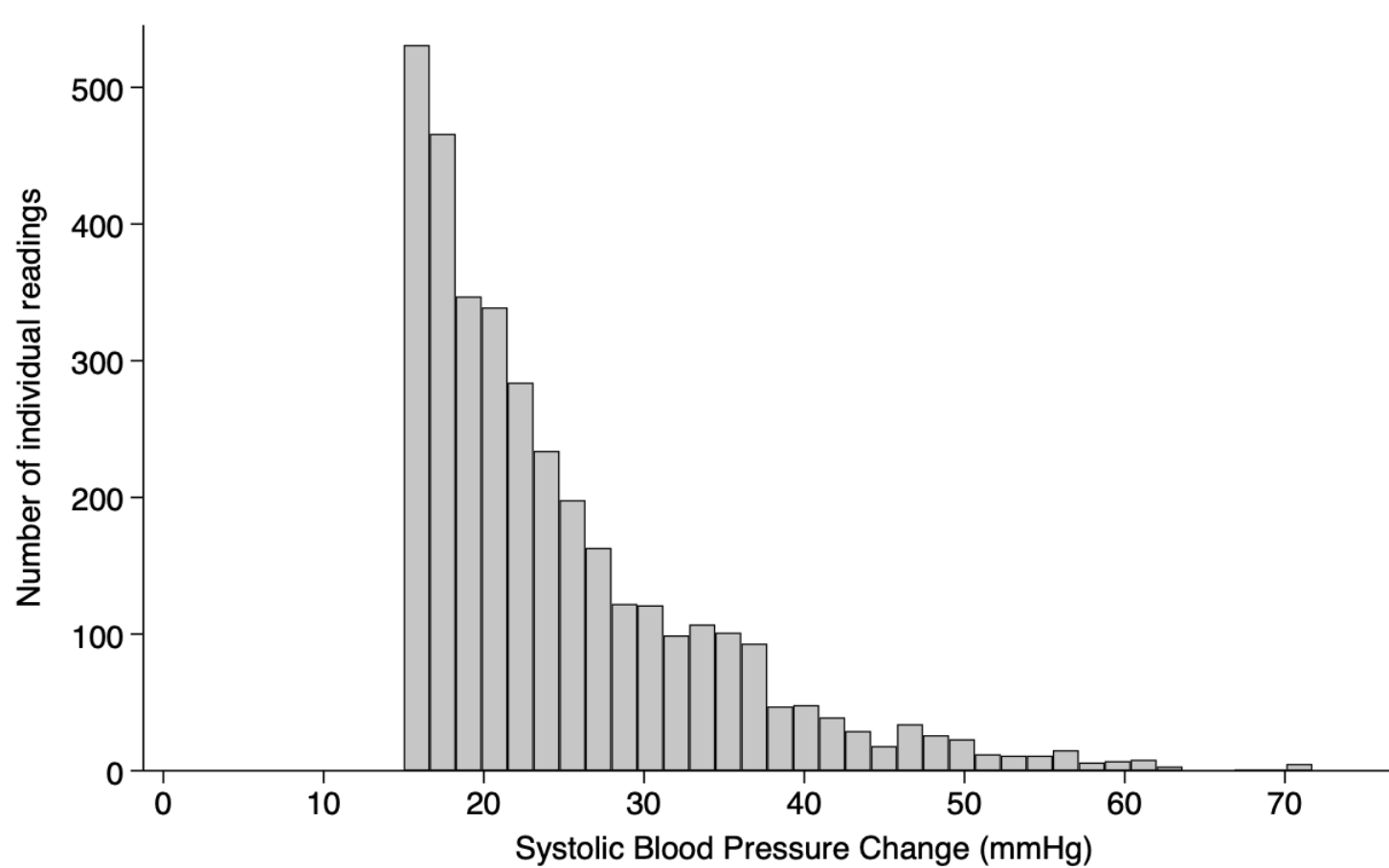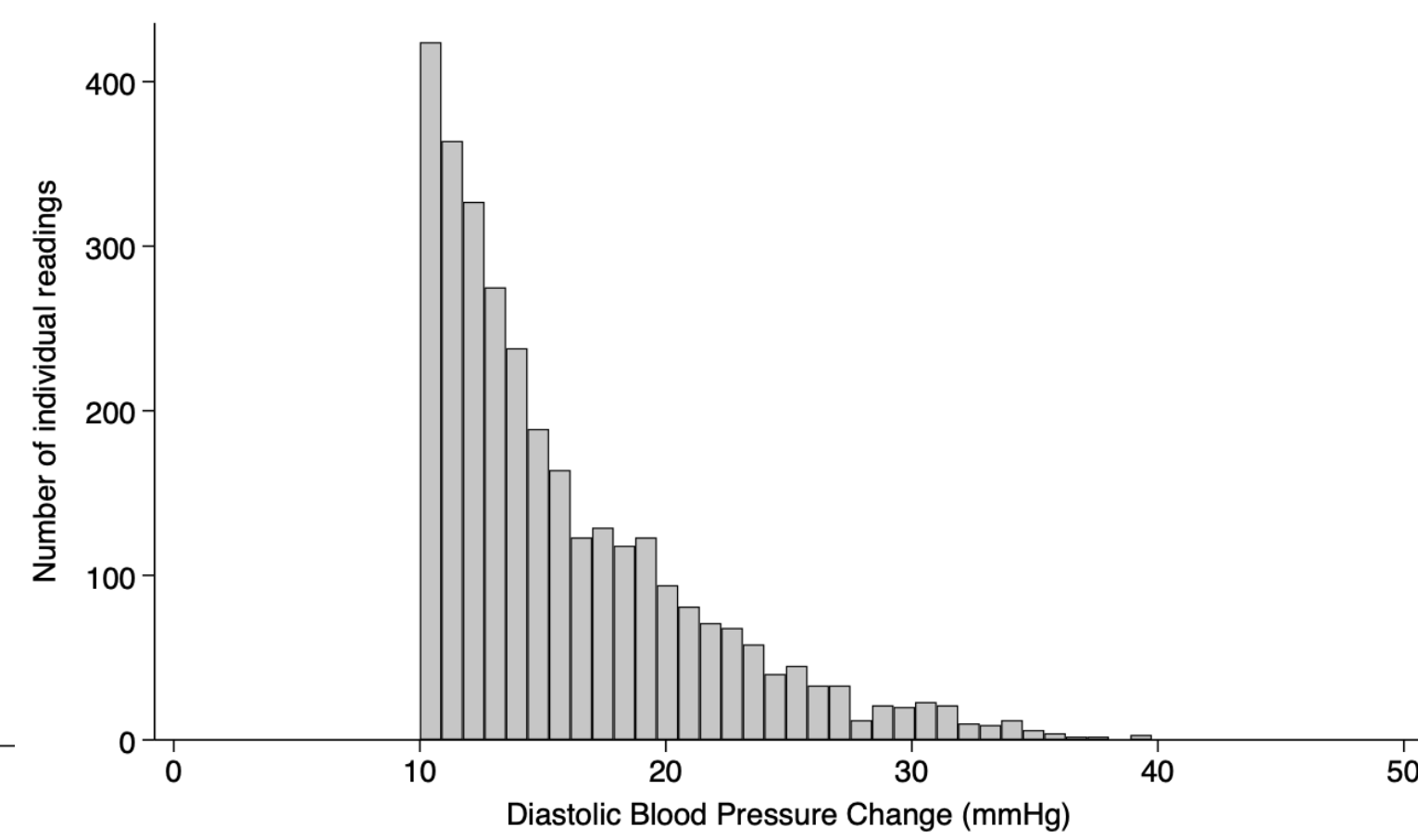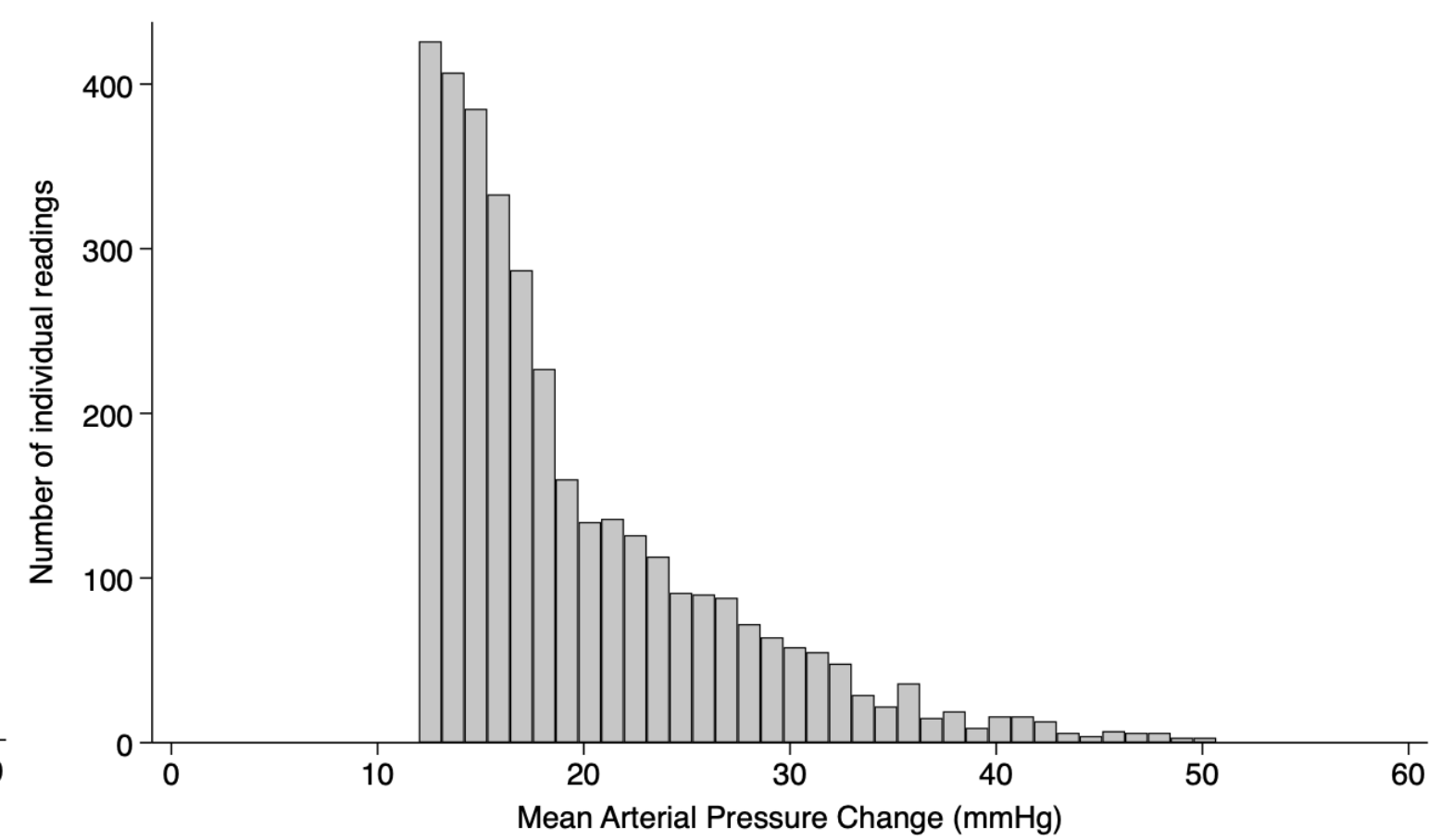

Supplement: Supplementary Figure S11 — Histogram of all reference blood pressure change parameters (n = 3,549 for systolic, n = 3,142 for diastolic and n = 3,510 for mean arterial pressure) included in the ISO Change analysis for each blood pressure parameter. The reference blood pressure change parameter was calculated by taking the reported reference blood pressure measurement at the end of the blood pressure change minus the reported reference blood pressure measurement at the start of the blood pressure change. [file Image11.pdf]
